# Vibrational resonant inelastic X-ray scattering in liquid acetic acid: a ruler for molecular chain lengths

**DOI:** 10.1038/s41598-021-83248-3

**Published:** 2021-02-18

**Authors:** Viktoriia Savchenko, Iulia Emilia Brumboiu, Victor Kimberg, Michael Odelius, Pavel Krasnov, Ji-Cai Liu, Jan-Erik Rubensson, Olle Björneholm, Conny Såthe, Johan Gråsjö, Minjie Dong, Annette Pietzsch, Alexander Föhlisch, Thorsten Schmitt, Daniel McNally, Xingye Lu, Sergey P. Polyutov, Patrick Norman, Marcella Iannuzzi, Faris Gel’mukhanov, Victor Ekholm

**Affiliations:** 1 Department of Theoretical Chemistry and Biology, KTH Royal Institute of Technology, 10691 Stockholm, Sweden; 2 International Research Center of Spectroscopy and Quantum Chemistry—IRC SQC, Siberian Federal University, Krasnoyarsk, Russia 660041; 3 Kirensky Institute of Physics, Federal Research Center KSC SB RAS, Krasnoyarsk, Russia 660036; 4 Department of Chemistry, Korea Advanced Institute of Science and Technology, Daejeon, 34141 Korea; 5 Department of Physics, AlbaNova University Center, Stockholm University, 106 91 Stockholm, Sweden; 6 Department of Mathematics and Physics, North China Electric Power University, Beijing, 102206 China; 7 Department of Physics and Astronomy, Uppsala University, Box 516, 751 20 Uppsala, Sweden; 8 MAX IV Laboratory, Lund University, Box 118, 221 00 Lund, Sweden; 9 Institute for Methods and Instrumentation in Synchrotron Radiation Research FG-ISRR, Helmholtz-Zentrum Berlin für Materialien und Energie Albert-Einstein-Strasse 15, Berlin, 12489 Germany; 10 Institut für Physik und Astronomie, Universität Potsdam, Karl-Liebknecht-Strasse 24-25, 14476 Potsdam, Germany; 11 Swiss Light Source, Photon Science Division, Paul Scherrer Institut, 5232 Villigen PSI, Switzerland; 12 Physical Chemistry Institute, University of Zürich, 8057 Zurich, Switzerland; 13 Department of Medicinal Chemistry, Uppsala University, Box 574, 75123 Uppsala, Sweden

**Keywords:** Molecular dynamics, Chemical physics, Atomic and molecular interactions with photons, Chemical physics, Structure of solids and liquids

## Abstract

Quenching of vibrational excitations in resonant inelastic X-ray scattering (RIXS) spectra of liquid acetic acid is observed. At the oxygen core resonance associated with localized excitations at the O–H bond, the spectra lack the typical progression of vibrational excitations observed in RIXS spectra of comparable systems. We interpret this phenomenon as due to strong rehybridization of the unoccupied molecular orbitals as a result of hydrogen bonding, which however cannot be observed in x-ray absorption but only by means of RIXS. This allows us to address the molecular structure of the liquid, and to determine a lower limit for the average molecular chain length.

## Introduction

The hydrogen bond (HB) is of central importance in chemistry and biochemistry and it is crucial in chemical reactions, supramolecular structures, molecular assemblies, and even life processes. Consequently, HBs have been immensely studied over the years, exploiting a plethora of spectroscopy and scattering methods, with liquid water as an important showcase^1^. The new generation synchrotron radiation sources have allowed for a refinement of the resonant inelastic X-rays scattering (RIXS) technique, which now gives access to detailed information about the nature of the HB, not only in liquid water^2–6^, but also liquids like acetone^7^ and methanol^8^.

Vibrational excitations observed in RIXS spectra give information about the local potential surface of the electronic ground state^9^, and about the electronic-vibronic dynamics during the scattering process. The dynamics is especially dramatic when the intermediate core-excited state is dissociative in a bond to a light atom, e.g., a state in which an O–H antibonding orbital is populated. This dynamics is sensitively reflected in extended vibrational progressions in RIXS spectra, and the exploration of these phenomena for the investigation of HBs in the liquid phase is a new research field which we are currently entering.

**Figure 1:**
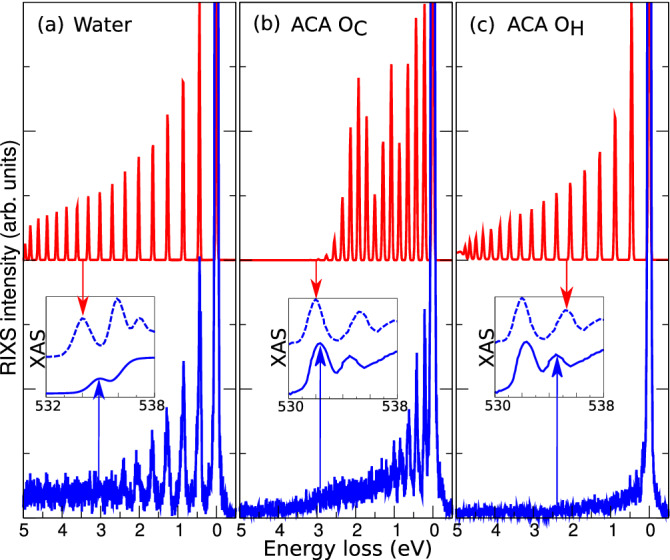
Liquid phase and gas phase RIXS spectra of water^5^ (**a**) and acetic acid (**b**, **c**). The experimental spectra of liquid phase (blue) are compared to computed gas phase spectra (red), obtained using *ab initio* methods as outlined in the text and normalized to $v=1$. The inserts shows the experimental XAS spectra of gas phase (dashed blue) water^5^ (**a**), acetic acid^28^ (**b**, **c**) and liquid (blue) water^5^ (**a**), acetic acid^29^ (**b**,**c**), where the vertical arrows point out the corresponding core-excitation energy in XAS. (In gas phase, on top of the resonances (red arrows). In (**a**) the excitation energy for liquid water is 535.0 eV. In (**b**, **c**) the excitation energies for liquid ACA are 532.1 and 534.6 eV for the $O_C$ $1s\rightarrow \pi ^*$ and $O_H$ $1s\rightarrow \pi ^*$ resonances, respectively). The RIXS intensities of ACA in panels (**b**) and (**c**) are matched to each other, as it is shown in Fig. S5^18^. The energy loss is the difference between the energies of incoming and emitted X-ray photons.

**Figure 2:**
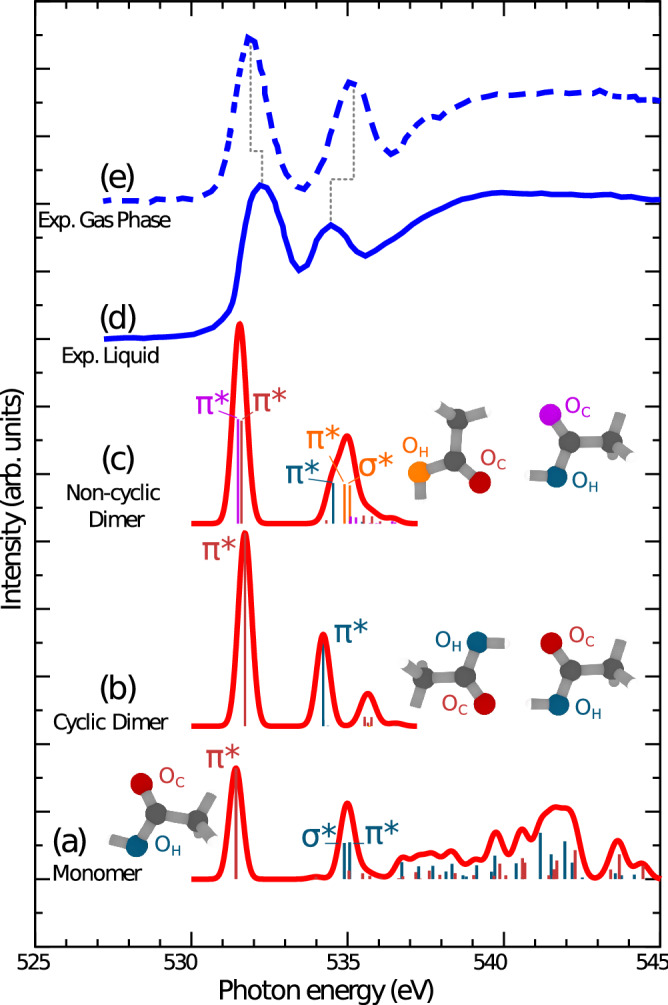
CVS-ADC(2)-x calculated XAS spectra of (**a**) the acetic acid monomer, (**b**) regular (cyclic) dimer and (**c**) inverted (non-cyclic) dimer, showed in comparison to measured XAS spectra of (**d**) acetic acid liquid from Ref.^29^ and (**e**) gas phase from Ref.^28^. The experimental and theoretical XAS spectra are shown in blue and red, respectively. The calculated oscillator strengths are shown as bar graphs, where each bar is coloured according to the atom of origin depicted in the molecular structure next to each spectrum. The XAS spectra have been obtained by broadening the bar graphs using Gaussian functions of 0.5 eV full width at half maximum. The high photon energy region (537 eV and above) of the dimer spectra is missing. This is due to the high computational cost of the CVS-ADC(2)-x method, where for the dimers we could only compute a limited number of excitation vectors, allowing the full description of only the first two experimental XAS peaks. In the case of the monomer, we could instead include enough excitation vectors to cover the full photon energy window used in the experiment.

Recently, unambiguous experimental evidence for ultrafast proton-transfer dynamics following excitation on the “pre-peak” resonance of liquid water was provided^5,6^. Significant nuclear rearrangement could be expected as the pre-peak resonance is associated with the $1s^{-1}4a_1$ excitation in gas-phase water, in which ultrafast dissociation arises from the antibonding O–H $\sigma ^*$ character of the $4a_1$ orbital^10^. RIXS spectra of liquid water excited at this resonance show a long vibrational progression (Fig. 1a). Also in corresponding measurements on methanol^8^ and acetone^7^, it was found that the long vibrational progression of the RIXS spectra of the free molecules was largely preserved in the liquid. These observations suggest that the intermolecular interactions, albeit significant, only play a minor role for the RIXS dynamics. Here we will demonstrate that this by no means is a general rule.

## Results

In the present work we combine state-of-the-art theory with high-resolution RIXS spectra (Fig. 1b,c) of liquid acetic acid (ACA) to analyze the influence of intermolecular interactions. We show that the HB has a dramatic influence on the molecular dynamics. Whereas ultrafast dissociation and accompanying extended vibrational progression are expected in RIXS spectra excited at the O–H antibonding intermediate state of O $1s^{-1}\sigma ^*$ character for the free molecule, the corresponding dynamics is almost entirely quenched in liquid ACA.

Carboxylic acids have received a lot of experimental and theoretical interest over more than a century. There is now a consensus that the predominant structures of ACA in the gas phase are the monomer and the symmetric cyclic dimer^11^. In the solid state, the crystalline unit cell consists of non-symmetric dimers which belong to a chain structure^11^. The local structure of liquid ACA is more complex and is still heavily debated (see Ref.^11–14^ and references therein). The linear cis-cis and cis-trans configurations are theoretically found in less ordered chains and rings of liquid acetic acid^11^. However, neutron scattering^15^ and Raman spectroscopy^16^ show a remarkable similarity in the hydrogen bond length when going from the crystal to the liquid structure and it has been concluded that the liquid structure is similar to that of a disordered crystal with predominant chain structures.

The interpretation of spectroscopic and scattering data of disordered liquids is complicated by the difficulty to get direct access to the structure, mainly because of the ambiguity in the analysis and interpretation of structural measurements. A crucial role is played by theoretical modeling, but in spite of theoretical support, the solution of the inverse problem is ambiguous (Refs.^12,14,15^). Therefore, complementary investigations are called for, and here we use modeling-supported X-ray absorption spectroscopy (XAS) and RIXS spectroscopy to shed light on the structure and interactions in ACA.

**Figure 3:**
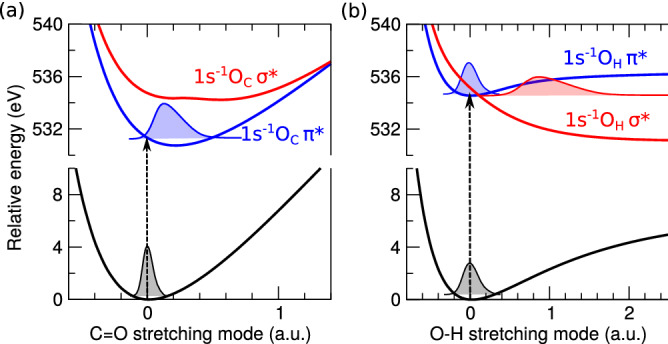
The potential energy curves from RASPT2 calculations of acetic acid in the ground, $1s^{-1}_{O_C}$ (a) and $1s^{-1}_{O_H}$ (b) core-excited states along the C=O and O–H stretching normal modes, respectively. Only the lowest bound $1s^{-1}_{O_C}\pi ^*$ contributes to the first XAS peak, and both close lying bound $1s^{-1}_{O_H}\pi ^*$ and dissociative $1s^{-1}_{O_H}\sigma ^*$ states form the second XAS peak of acetic acid monomer (Fig. 2). The oscillator strengths are almost the same for the $1s^{-1}_{O_H}$ core-excited states and they are about four times smaller as compared to the $1s_{O_C}\rightarrow \pi ^*$ core-excitation (Fig. 2).

**Figure 4:**
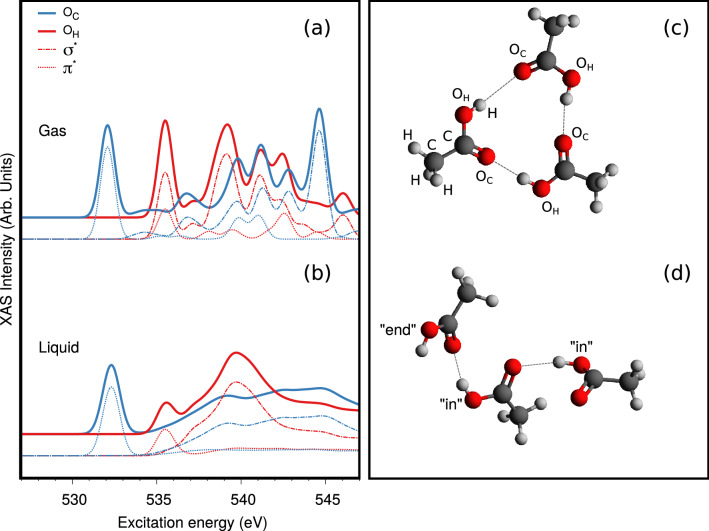
XAS from DFT calculations of the $O_C$ (blue) and $O_H$ (red) K-edges in gas phase (**a**) and liquid (**b**) ACA are shown and further decomposed into out-of-plane ($\pi ^*$-type, dotted) and in-plane ($\sigma ^*$-type, dashed-dotted) contributions. The spectra are computed using the XFH transition potential method and AIMD approach (see for more details the Supplementary information^18^). (**c**) A cyclic structure without “end” OH groups. (**d**) A chain structure of liquid acetic acid which contains an “end” OH group.

The spectral region of our interest consists of the first two peaks in the oxygen K-edge XAS of acetic acid (Fig. 2). Because of the different chemical shifts of the *O* 1*s* levels of carbonyl ($O_C$) and hydroxyl ($O_H$) oxygen atoms the chemical site can be selected by tuning the excitation energy. Indeed, the XAS spectrum of the acetic acid monomer has two distinct pre-edge peaks and the analysis shows that each peak is related to a distinct oxygen atom (Fig. 2). XAS simulations of the isolated ACA show that the first peak around 532 eV is due to $O_C$ $1s\rightarrow \pi ^*$ core excitation, while the second peak at 535 eV is formed by two close-lying peaks $O_H$ $1s\rightarrow \pi ^*$ and $O_H$ $1s\rightarrow \sigma ^*$ with almost the same intensity^17^. The C–O equilibrium distance of the ground state and the core excited state $O_C$ $1s^{-1}\pi ^*$ state differs (Fig. 3) due to the strong interaction between the 2*p* orbitals of carbon and oxygen. Consequently, a long C=O vibrational progression is predicted for the gas-phase RIXS spectrum (Fig. 1b).

The O–H equilibrium distance of the core excited $O_H$ $1s^{-1}\pi ^*$ state of the ACA monomer is almost the same as that of the ground state, largely because the oxygen 2*p* orbital does not interact with the hydrogen 1*s* orbital (Fig. 3), and consequently, RIXS spectra excited at this resonance are predicted to show almost no OH vibrations (Fig. S3^18^). In contrast, the $O_H$ $1s^{-1}\sigma ^*$ is dissociative (Fig. 3), resulting in an extensive vibrational progression (Fig. 1c) in the corresponding monomer RIXS spectrum.

Experimentally, we find that the vibrational progressions predicted for free molecules to a large extent are preserved in the RIXS spectra of the liquids. The sensitivity to the chemical surrounding is typically accentuated for highly excited vibrational states, where potential surfaces are modified by intermolecular interactions. Therefore, vibrational progressions are often smeared out and attenuated at large energy losses. For lower energy losses, on the other hand, the behavior simulates gas-phase predictions. This is illustrated for the *O* $1s\rightarrow 4a_1$ resonance in water (Fig. 1a), and for the $O_C$ $1s\rightarrow \pi ^*$ resonance in ACA (Fig. 1b). The predicted O–H vibrations for gas-phase water and O-C vibrations for ACA, are both retained in the RIXS spectra of the liquids, at least up to $v=5$, before intermolecular interactions smear out the intensity.

For the second resonance of liquid ACA the situation is dramatically different (Fig. 1c). Whereas a long progression of O–H stretch vibrations with a $\omega _{\mathrm{vib}}^{\mathrm{exp}} \approx$ 0.44 eV is predicted for the free molecule, vibrational excitations are virtually absent in the experimental RIXS spectrum. This observation suggests that intermolecular interactions dramatically quench the ultrafast dissociation, and that the HB has a major impact on the dynamics. Notable, however, is a weak vibrational peak structure, where the vibrational peaks are separated 0.16-0.17 eV. These could be attributed to the OH bending mode and/or carboxyl C–O stretch^19,20^. To shed light on the unusual effect of complete quenching of the O–H stretch progression, we analyze the core-excitations of ACA molecules in gas and liquid phases. For comparison with liquid phase, an experimental gas-phase spectrum would be ideal. However, the spectral quality needed to make a comparison is state-of-the-art, and presently there is no instrument available where such a gas-phase measurement can be done. Instead, we use a high-quality theoretical calculation of the RIXS spectrum of the ACA monomer (Fig. 1c) which is expected to accurately predict an experimental gas-phase spectrum (compare to our previous study of RIXS of free water molecule^21^). We use this as a reference to compare with the measured RIXS spectra of the liquid.

We represent the gas-phase ACA by the monomer and assume that a chain of hydrogen bonded molecules is the representative structure of liquid ACA (Fig. 4d). Here, we identify two distinct OH groups: one at the end of the chain, not involved in HB, which we denote “end”, and one inside the chain, involved in HB, which we denote “in”. These two OH groups are expected to have significantly different XAS spectra, which we have computed using the shortest possible chains, i.e. the dimers. Two types of dimers are included, namely the regular dimer (centrosymmetric cyclic dimer, which also exist in the gas phase) and the inverted dimer (asymmetric noncyclic dimer which is the shortest representative of the chain present in the liquid). The XAS spectra, computed in the same way as for the monomer, are shown in Fig. 2.

## Discussion

Note that while $\pi ^*$ and $\sigma ^*$ resonances are predicted to contribute almost equally to the 535-eV peak in the XAS spectrum of the monomer (Fig. 2a), the $\sigma ^*$ resonance *does not contribute at all* in the regular dimer, as previously discussed in the literature^17^ (Fig. 2b). This dramatic change is an obvious effect of the HB, which affects both “in” OH groups in the regular dimer. For the inverted dimer the XAS spectrum comprises transitions in both “in” and “end” OH groups (Fig. 2c), and whereas the “in” OH group has similar features as the OH groups in the regular dimer the XAS signal of the “end” OH resembles the signal of the OH group in the ACA monomer. This is a manifestation of the building block principle, according to which the occurrence of specific groups leaves a fingerprint in the spectrum. The effect of the HBs is, however, not very large in the total XAS, and condensation results only in slight shifts of the spectral features (Fig. 2d–e). In contrast, a strong effect is expected in RIXS: Whereas the “end” OH groups are almost unaffected by the HB, and because the 535-eV peak comprises transitions to the dissociative $\sigma ^*$ core-excited state, a long vibrational progression is expected in the RIXS spectrum, the peak in the spectra of the “in” OH groups has no contribution from the $\sigma ^*$ state, and consequently no significant vibrational excitations are expected. In this way a RIXS measurement allows us to clearly distinguish between “in” and “end” OH groups, and the observation that vibrational excitations are virtually absent shows that HBs are significant, and puts an upper limit to the occurrence of “end” OH groups in the liquid, which we estimate in the following.

The total number of ACA units in a liquid can be written approximately as

1 $$\begin{aligned} {{\mathcal {N}}}=\ell _{\mathrm{ch}}N_{ch}+\ell _{\mathrm{cycl}}N_{\mathrm{cycl}} \end{aligned}$$

Here $\ell _{\mathrm{ch}}$ and $\ell _{\mathrm{cycl}}$ are the average number of ACA monomers (or length in ACA units) in chain and cyclic structures, respectively, while $N_{ch}$ and $N_{cycl}$ are the average number of chains and cyclic structures, respectively. Here we assume that the chain is the representative chain structure from crystalline ACA with one “end” OH group (see Fig. 4d). The cyclic structures have only “in” OH groups (see Fig. 4c). The relative abundance of the “end” OH groups is $N_{ch}/{{\mathcal {N}}}$. This ratio represents the relative intensity of the RIXS profile with the OH vibrational progression caused by the “end” OH group.

A detailed analysis of peak-to-noise ratio in the RIXS spectra of liquid ACA and the calculated $v=1$ peak intensities in the ACA monomer is performed in the Supplementary information^18^. From this analysis, we conclude that for a vibrational progression to exceed the experimental noise level (more precisely the level of detectability), the fraction of chains must obey the following relation ${{\mathcal {N}}}/N_{\mathrm{ch}}=\ell _{\mathrm{eff}}=\ell _{\mathrm{ch}}+\ell _{\mathrm{cycl}}N_{\mathrm{cycl}} /N_{\mathrm{ch}} \lesssim 3.5$, where $\ell _{\mathrm{eff}}$ denotes the effective length of chain or cycle unit.

Since our measurements do not display a OH vibrational progression, this means that the effective length $\ell _{\mathrm{eff}}$ should be longer than 3.5 monomer units.

2 $$\begin{aligned} \frac{{{\mathcal {N}}}}{N_{\mathrm{ch}}}=\ell _{\mathrm{ch}}+\ell _{\mathrm{cycl}}\frac{N_{\mathrm{cycl}} }{N_{\mathrm{ch}}} > 3.5. \end{aligned}$$

Let us take the recommended structure from a recent analysis^15^ of neutron scattering data, where it is claimed that the dominant structure is the trimer chain. If trimer chains were the only motif then $N_{\mathrm{cycl}}=0$ and $\ell _{\mathrm{ch}}=3$, so that the condition for observing vibrational excitation would be fulfilled. Our results indicate that the effective chain length is longer. Our estimate corresponds to a lower bound for $\ell _{\mathrm{eff}}$ within the current *signal*/*noise* ratio. One can expect to refine the estimate, possibly giving an increased $\ell _{\mathrm{eff}}$, in future experiments with improved statistics.

So far, we have discussed RIXS only from the perspective of the $O_H$ $1s^{-1}\sigma ^*$ core-excited state of the ACA monomer and dimers. However, one may also expect vibrational structure in RIXS due to the $O_H$ $1s^{-1}\pi ^*$ core-excited states which are also populated when the photon energy is tuned near the second XAS peak (Fig. 2). One cannot exclude that the HB changes the potential energy curve of the $\pi ^*$ core-excited states and opens inelastic scattering channels, but, as shown in the Supplementary information^18^, this does not occur.

Additionally, ab initio molecular dynamics (AIMD) simulations of the XAS spectrum were performed to investigate the sensitivity to solvation and HBs, using the transition-potential DFT approach (see the Supplementary information^18^). The results of these simulations (Fig. 4b) confirm that the $\sigma ^*$ core-excited state only contributes little to the second XAS peak in liquid ACA which is dominated by a single $\pi ^*$ core-excited state. The individual contributions of the C=O and O–H oxygen atoms to the *O* 1*s* XAS spectra (displayed in blue and red lines, respectively) show that liquid interactions have a profoundly different influence on the two sets of core-excited states. In Fig. 4a,b, we also show the decomposition of the XAS spectrum contributions into in-plane and out-of-plane components which allows us to understand the influence of the HB on the O–H $\sigma ^*$ core-excited state. In Fig. S1b,c of the Supplementary information^18^ we further investigate the hydrogen bonding by ordering the out-of-plane and in-plane contributions according to hydrogen bond distance in the donating direction of the OH group, and we identify the trend that the $\sigma ^*$ character only barely reaches down to the O–H pre-edge peak even for very distorted hydrogen bonding configurations.

In conclusion, our RIXS measurements of liquid ACA show the absence of a vibrational progression of the OH stretch mode under pre-edge core-excitation of the hydroxyl oxygen contrary to liquid water and methanol. We attribute this behavior to an unusually strong influence of the HB, which changes the character of the intermediate state and quenches the vibrational progression. The effect allows us to put an upper limit to the average concentration of “end” OH groups in the liquid that are not strongly involved in HBs, implying that the average length of chains must be larger than 3 and/or there must be a significant abundance of cyclic structures. Finally, we note that the method exploited here, based on the quenching of the vibrational progression in RIXS, can be used for structure investigations with some generality: We have found that the intensity of the OH vibrational progression can be used as a “ruler” of chain length. X-ray diffraction^22^ shows that the ratio between dimers and chains is shifted towards dimers in the case of liquid propionic acids compared to ACA, and the proposed structure may be validated with this new method. We envision applications to a variety of liquids, such as formic acid, methyl acetate, and methyl propionate in the near future.

## Methods

### Experiment

The experiment was performed at room temperature with the SAXES spectrometer^23^ at the RIXS end station of the ADRESS beam line^24^ at the Swiss Light Source at the Paul Scherrer Institut. The liquid acetic acid acquired from Sigma-Aldrich had a purity level $\ge$99% and the water sample was Milli-Q purified and de-ionized. We utilized a flow-cell separating the sample from the vacuum by a 100 nm thick $\hbox {Si}_3$ $\hbox {N}_4$ window. Due to the risk of window rupture under irradiation, the cell was moved every 5 min. The resonantly scattered photons were detected at a 90$^{\circ }$ angle from the incoming horizontally polarized X-ray beam by three different detectors with an experimental resolution of $\approx$45 meV for liquid ACA and water. The spectra were measured at several times (3-6 times) at each excitation energy where the duration of each measurement was 5 minutes. The presented spectra were calculated as the sum of these spectra and normalized by dividing by the number of measurements times the number of detectors. To avoid errors from this procedure, the spectra of these individual scans were shifted to same energy scale by using a fit to the elastic line before joining them for further data processing. The energy calibration was based on the $\hbox {O}_2$ RIXS spectrum^25^. Moreover, the RIXS intensity integrated in a broad energy range (up to 12 eV energy loss) was monitored in order to confirm peaks of the absorption resonances (see Fig. S6). We compare the intensities of RIXS spectra for the scattering through the carbonyl ($O_C$) and hydroxyl ($O_H$) oxygen atoms. The normalization of intensities of these scattering channels was refined by adjusting the spectral intensity to achieve the noise (by the root mean square error) in the signal-free anti-Stokes range of − 6 to − 2 eV to be equal for these spectra (See Supplementary information^18^ and Fig. S5).

### Theory

The main part of simulations were performed using post Hartree–Fock ab initio methods accompanied by the techniques based on density functional theory (DFT) and AIMD. We computed the X-ray absorption profile of the ACA monomer and dimers (Fig. 2) using the core-valence separation (CVS) approximation at the algebraic diagrammatic construction (ADC) level of theory. Specifically, the CVS-ADC(2)-x variant was used in combination with the 6-31++G(p,d) basis set, as outlined in Refs.^8,26^. It is noted that the XAS profile of the ACA monomer as computed using the multi-reference RASPT2 approach displays a good agreement with the ADC(2)-x and TDDFT simulations (Fig. S2^18^). The RIXS profiles (Figs. 1, S3^18^) were computed using the wave packet software as described in Refs.^6,8,21,27^ using the potential energy curves (PECs) along the O–H and C–O stretching modes. The ground and core-excited PECs (Fig. 3) were determined at the RASSCF/ANO-RCC-VTZP level (with 10 electrons in 13 active molecular orbitals) followed by RASPT2 calculations to account for dynamic electron correlation effects. Finally, additional simulations of XAS spectra using AIMD were performed (see Figs. 4, S1^18^). The full computational details can be found in the Supplementary information^18^.

## Supplementary information

Supplementary information.
