# Supplementary material for: Vibrational resonant inelastic X-ray scattering in liquid acetic acid: a ruler for molecular chain lengths
Source: Sci Rep. 2021 Feb 18;11:4098. doi: 10.1038/s41598-021-83248-3 (PMC7893077; doi:10.1038/s41598-021-83248-3)
Supplement: Supplementary file 1 — Supplementary information. [file 41598_2021_83248_MOESM1_ESM.pdf]

Supplementary information of article Vibrational resonant inelastic X-ray scattering in liquid acetic acid: a ruler for molecular chain lengths

Viktoriia Savchenko[1,2,3,*], Iulia Emilia Brumboiu[1,4], Victor Kimberg[1,2,3,*], Michael Odelius[5,*], Pavel Krasnov[2,3], Ji-Cai Liu[6], Jan-Erik Rubensson[7], Olle Björneholm[7], Conny Såthe[8], Johan Gråsjö[7,13], Minjie Dong[7], Annette Pietzsch[9], Alexander Föhlisch[9,10], Thorsten Schmitt[11], Daniel McNally[11], Xingye Lu[11], Sergey P. Polyutov[2,3], Patrick Norman[1], Marcella Iannuzzi[12], Faris Gel'mukhanov[1,2,3], and Victor Ekholm[7,8]

[1]Department of Theoretical Chemistry and Biology, KTH Royal Institute of Technology, Stockholm, 10691, Sweden
[2]International Research Center of Spectroscopy and Quantum Chemistry - IRC SQC, Siberian Federal University, Krasnoyarsk, 660041, Russia
[3]Kirensky Institute of Physics, Federal Research Center KSC SB RAS, Krasnoyarsk, 660036, Russia
[4]Department of Chemistry, Korea Advanced Institute of Science and Technology, Daejeon, 34141, Korea
[5]Department of Physics, AlbaNova University Center, Stockholm University, Stockholm, SE-106 91, Sweden
[6]Department of Mathematics and Physics, North China Electric Power University, Beijing, 102206, China
[7]Department of Physics and Astronomy, Uppsala University, Box 516, Uppsala, SE-751 20, Sweden
[8]MAX IV Laboratory, Lund University, Box 118, Lund, SE-221 00, Sweden
[9]Institute for Methods and Instrumentation in Synchrotron Radiation Research FG-ISRR, Helmholtz-Zentrum Berlin für Materialien und Energie Albert-Einstein-Strasse 15, Berlin, 12489, Germany
[10]Institut für Physik und Astronomie, Universität Potsdam, Karl-Liebknecht-Strasse 24-25, Potsdam, 14476, Germany
[11]Swiss Light Source, Photon Science Division, Paul Scherrer Institut, Villigen PSI, CH-5232, Switzerland
[12]Physical Chemistry Institute, University of Zürich, Zürich, 8057, Switzerland
[13]Department of Medicinal Chemistry, Uppsala University, Box 574, Uppsala, 75123, Sweden
[*]Correspondence and requests for materials should be addressed to VS (viksav@kth.se), VK (kimberg@kth.se), and MO (odelius@fysik.su.se).

Details of *ab initio* molecular dynamics simulations and sampling of XAS spectra

Ab initio molecular dynamics (AIMD) simulations were performed on the level of density functional theory in the CP2K software suite (version 6.0)[1–4] using a dispersion corrected BLYP functional[5–8]. The Gaussian and Plane Wave (GPW) method[4] was employed in combination with GTH pseudo-potentials[9–11] with Gaussian basis sets (TZVP-GTH)[12] to describe the Kohn-Sham orbitals and an auxiliary plane wave basis (with a cut-off of 400 Ry) to describe the electron density. The AIMD simulations were initialized from a 1 ns classical molecular dynamics simulation in the MDynaMix code based on the OPLS-AA force field[13,14]. Simulations were performed at the experimental density (ρ=1.05 g/cm^3) in the NVT ensemble[15] with a time-step of 0.5 fs. After 10 ps equilibration, the systems were sampled for 40 ps. For comparison, the optimized isolated molecule was studied in a cubic simulation cell of dimension 30 Å, in combination with a Poisson solver to model isolated systems[16].

Simulated oxygen K-edge (O1*s*) X-ray absorption spectra were derived from DFT transition potential (TP) self-consistent field calculations using the excited-state full core-hole (XFH)[17] method as implemented in the Gaussian augmented plane wave (GAPW) method[18] in the CP2K software suite (version 6.0)[1–4] using an all-electron description with the 6-311++G** basis set[19,20] and a 400 Ry cut-off for the plane wave expansion of the electron density. For convergence of the transition potential wave function a smearing procedure with an electronic temperature of 3000 K was employed, due to problems to converge the XAS for the O_H core-excitation in XFH. Comparison against XFH without smearing confirmed that there is no significant effect on the quality of the spectra. Each discrete XAS spectrum was based on 2000 unoccupied Kohn-Sham orbitals in the TP calculation. The discrete transitions were convoluted with a Gaussian broadening with a full width half maximum (FWHM) of 1.0 eV and ad-hoc energy shifts were applied (XFH:-16.8 eV) for direct comparison to the measured spectra. The mean O1*s* XAS was sampled over 40 configurations, which allowed for configurational analysis by partitioning the configuration into different hydrogen environments. The results of the XAS simulations sampled over the AIMD simulation of liquid acetic acid

are presented in Fig. S1.

Details of *ab initio* calculations

The molecular structures of the acetic acid (ACA) monomer and two dimers were optimized in Gaussian 16[21], using the second order Møller-Plesset perturbation theory[22–27] and the cc-pVTZ basis set[28–30]. The geometry optimization of the dimers also included the counterpoise correction to limit the basis set superposition error[31,32]. The relaxed molecular structures were then used to compute X-ray absorption spectra at the algebraic diagrammatic construction (ADC) level of theory[33] as implemented in the Q-Chem 5.1 quantum chemistry software[34]. Specifically, the core-valence separation (CVS) approximation for the extended second order ADC variant, i.e. CVS-ADC(2)-x[35–37], was used in combination to the 6-311++G** basis set[19,38] to compute the excitation energies and transition dipole moments from O 1s core levels to unoccupied molecular orbitals. To facilitate the comparison to experiment, the oscillator strengths were broadened using Gaussian functions of 0.5 eV FWHM. In the case of the dimers, due to the high computational cost of the CVS-ADC(2)-x method, only 20 excitation vectors could be included and, therefore, the high photon energy window of the spectra (above 537 eV) is missing. No shift of the calculated XAS spectra was performed.

The computation of potential energy curves (PECs) of the ground and core-excited states of the ACA monomer was performed with scalar-relativistic restricted active space self consistent field (RASSCF) calculations (with 10 electrons in 13 active molecular orbitals, including one O 1s in RAS1) followed by perturbative (RASPT2) corrections to include dynamical correlation, as implemented in the MOLCAS code (version 8.2) using the ANO-RCC-VTZP basis set, similar to Ref.[39]. The weak geometry dependence of the transition dipole moment was neglected in the RIXS simulations, except for the case discussed in Figs. S2 and S3 of the next Section.

Vibrational structure of RIXS through the π^* core-excited states

We discussed in the main text mostly the σ^* RIXS channel and related quenching of the vibrational progression in liquid ACA where the dominant structure is the chain of hydrogen bonds. Here we focus on the π^* RIXS channel in the shortest possible chain, i.e. the inverted dimer. Fig. 2 of the main text shows that two $O_H 1s^{-1}\pi^*$ core excited states contribute to the second XAS peak. The first one is related to the "in" oxygen atom while the second one is due to the core-excitation of the "end" oxygen. It is not surprising that the RIXS profile through the core-excitation of the "end" oxygen has no vibrational structure because PEC of this core-excited state is almost parallel to the ground state PEC, similar to the ACA monomer (see Fig. 3 of the main text).

However, the "in" O_H atom participates in the hydrogen bond with the O_C atom. Therefore, one can expect that the hydrogen bond, affecting the OH PEC in the $O_H 1s^{-1}\pi^*$ state, may result in a vibrational progression of RIXS. Firstly, we computed the $O_H 1s^{-1}\pi^*$ PECs (Fig. S2) using the TDDFT B3LYP/aug-cc-pVTZ method [29,40,41] including the Resolution of the Identity (RI) approximation[42–47] and auxiliary aug-cc-pVTZ/C basis set[48] in the ORCA software[49,50].

To check the TDDFT results we used also higher level simulations based on the CVS-ADC(2)-x technique (Fig. S2). The TDDFT method displays two close-lying diabatic π^* core-excited states of the "in" oxygen which cross each other. Fig. S2 shows the related adiabatic states and corresponding oscillator strengths (f) with strong dependence on the elongation of the OH bond. We computed the RIXS profile for the lowest adiabatic core-excited state taking into account the R-dependence of the transition dipole moment $d \propto \sqrt{f}$. The simulation (Fig. S3) shows that the corresponding RIXS profile through this core-excited state has no significant vibrational structure. The RIXS through the second π^* core excited state gives negligible contribution because of the small transition dipole moment.

The higher level CVS-ADC(2)-x calculations show only one π^* core-excited state in the region of our interest. The absence of the avoided crossing with another possible state is seen clearly from the absence of significant R-dependence of the oscillator strength (see Fig. S2). The CVS-ADC(2)-x PEC is more shallow in comparison with the TDDFT calculation. However, the CVS-ADC(2)-x approach, similar to the TDDFT method, gives a very small shift of the minimum of the PEC with respect to the ground state equilibrium. This explains the absence of any significant vibrational structure in the simulated RIXS spectrum (Fig. S3).

Detailed investigation of detectability of a vibrational RIXS signal at the O_H pre-edge resonance of liquid ACA

To investigate the minimum peak to noise ratio required for a peak to be detectable by eye, a series of peak to noise ratio simulations were made. RIXS spectra measured at five excitation energies near the top of the O_H pre-edge resonance (534.1, 534.4, 534.7, 535.0, 535.3 eV) were averaged in order to improve statistics and superimposed with a Gaussian function of FWHM=0.05 eV centered at 0.44 eV energy loss to mimic the $\nu_{OH} = 1$ vibrational resonance (see Fig. S4). The peak width was chosen corresponding to the peak width of the other vibrational peaks in measured spectra. The peak intensity A_0 of the

Gaussian function was varied from 0.0 to 0.7 (see legends in Fig. S4). We judge that the peak is distinguishable by eye already at the peak intensity $A_0 = 0.2$.

In Fig. S5, the zoom-in of the RIXS features of liquid ACA shows the relative strengths of the vibrational progression at the O_C pre-edge resonance and the O_H pre-edge resonance, and it is clear that the core-excitation at the O_H pre-edge resonance only gives rise to vibrational excitation of low-energy vibrational modes. We notice the $v = 1$ peak in the RIXS at the O_C pre-edge resonance has a peak intensity of σ_{OC}^{01} =6.3 at the current normalization as it is described in the main text. The peak intensity was obtained by removing the contribution from the background of the elastic peak. For the analysis of the expected intensity of a O-H vibrational progression RIXS at the O_H pre-edge resonance σ_{OH}^{01}, we can use the experimental observation that σ_{OC}^{01} =6.3 in liquid ACA together with theoretical results for the two different resonances in gas phase ACA.

Assuming that the peak intensities σ_{OC}^{01} and σ_{OH}^{01} are proportional to the transition dipole moment to the power of 4 ($d^4_{1s_{OC}\pi^*}$ and $d^4_{1s_{OH}\sigma^*}$) and assuming Franck-Condon factors (FC_{OC}^{01} and FC_{OH}^{01}) with the same proportionality constant ζ for both the O_H and O_C pre-edge resonances

$$\begin{aligned}\sigma_{OC}^{01} &= 6.3 = \zeta \times d^4_{1s_{OC}\pi^*} FC_{OC}^{01}, \\ \sigma_{OH}^{01} &= \zeta \times d^4_{1s_{OH}\sigma^*} FC_{OH}^{01} \\ &= 6.3 \left(\frac{d^4_{1s_{OH}\sigma^*}}{d^4_{1s_{OC}\pi^*}} \right) \frac{FC_{OH}^{01}}{FC_{OC}^{01}} \approx 0.7,\end{aligned} \tag{1}$$

given from our simulations that $FC_{OH}^{01}/FC_{OC}^{01} = 0.78$ and $(d_{1s_{OH}\sigma^*}/d_{1s_{OC}\pi^*})^2 = 1/2.65$. This gives an estimate for the peak intensity for ACA monomer in the gas phase. In the liquid, this corresponds to the ACA molecules with an "end" OH group of which there is only one in each chain. Total number of ACA monomers is

$$\mathcal{N} = N_{\text{ch}}\ell_{\text{ch}} + N_{\text{cycl}}\ell_{\text{cycl}} \tag{2}$$

Cycle structures have no "free" OH groups. Each chain structure has one "free" OH group. Thus the relative number of "free" OH groups is

$$\frac{N_{\text{ch}}}{\mathcal{N}}. \tag{3}$$

This means that signal should be reduced by this factor as

$$\text{signal(liquid)} = \sigma_{OH}^{01}\frac{N_{\text{ch}}}{\mathcal{N}} = \frac{\sigma_{OH}^{01}}{\ell_{\text{eff}}}, \tag{4}$$

where ℓ_{eff} is defined as

$$\ell_{\text{eff}} = \ell_{\text{ch}} + \frac{N_{\text{cycl}}}{N_{\text{ch}}}\ell_{\text{cycl}}. \tag{5}$$

Since we do not see any O-H vibrational structure in the experiment and following our analysis of noise to peak ratio (Fig. S4) we can conclude that peak intensity in liquid is smaller than 0.2

$$\text{signal(liquid)} = \frac{\sigma_{OH}^{01}}{\ell_{\text{eff}}} < 0.2, \tag{6}$$

which provides us with estimate for the effective length

$$\ell_{\text{eff}} \gtrsim 0.7/0.2 = 3.5. \tag{7}$$

This means that the effective length of the ACA structure should be comparable to or longer than 3.5.

References

1. CP2K version 6.0, the CP2K developers group. http://www.cp2k.org (2017).
2. Hutter, J., Iannuzzi, M., Schiffmann, F. & VandeVondele, J. Wiley Interdiscip. *Rev.: Comput. Mol. Sci* **4**, 15–25, DOI: 10.1002/wcms.1159 (2014).
3. VandeVondele, J. *et al.* Quickstep: Fast and accurate density functional calculations using a mixed Gaussian and plane waves approach. *Comput. Phys. Commun.* **167**, 103–128, DOI: https://doi.org/10.1016/j.cpc.2004.12.014 (2005).

4. Lippert, G., Parrinello, M. & Hutter, J. A hybrid Gaussian and plane wave density functional scheme. *Mol. Phys.* **92**, 477–488, DOI: https://doi.org/10.1080/002689797170220 (1997).

5. Becke, A. D. Density-functional exchange-energy approximation with correct asymptotic behavior. *Phys. Rev. A* **38**, 3098–3100, DOI: 10.1103/PhysRevA.38.3098 (1988).

6. Lee, C., Yang, W. & Parr, R. G. Development of the Colle-Salvetti correlation-energy formula into a functional of the electron density. *Phys. Rev. B* **37**, 785, DOI: https://doi.org/10.1103/PhysRevB.37.785 (1988).

7. Grimme, S., Ehrlich, S. & Goerigk, L. Effect of the damping function in dispersion corrected density functional theory. *J. Comput. Chem.* **32**, 1456–1465, DOI: https://doi.org/10.1002/jcc.21759 (2011).

8. Grimme, S., Antony, J., Ehrlich, S. & Krieg, H. A consistent and accurate ab initio parametrization of density functional dispersion correction (DFT-D) for the 94 elements H-Pu. *J. Chem. Phys.* **132**, 154104, DOI: https://doi.org/10.1063/1.3382344 (2010).

9. Krack, M. Pseudopotentials for H to Kr optimized for gradient-corrected exchange-correlation functionals. *Theor. Chem. Accounts* **114**, 145–152, DOI: https://doi.org/10.1007/s00214-005-0655-y (2005).

10. Hartwigsen, C., Gœdecker, S. & Hutter, J. Relativistic separable dual-space Gaussian pseudopotentials from H to Rn. *Phys. Rev. B* **58**, 3641, DOI: https://doi.org/10.1103/PhysRevB.58.3641 (1998).

11. Goedecker, S., Teter, M. & Hutter, J. Separable dual-space Gaussian pseudopotentials. *Phys. Rev. B* **54**, 1703, DOI: https://doi.org/10.1103/PhysRevB.54.1703 (1996).

12. VandeVondele, J. & Hutter, J. Gaussian basis sets for accurate calculations on molecular systems in gas and condensed phases. *J. Chem. Phys.* **127**, 114105, DOI: https://doi.org/10.1063/1.2770708 (2007).

13. Lyubartsev, A. P. & Laaksonen, A. M.DynaMix–a scalable portable parallel MD simulation package for arbitrary molecular mixtures. *Comput. Phys. Commun.* **128**, 565–589, DOI: https://doi.org/10.1016/S0010-4655(99)00529-9 (2000).

14. Jorgensen, W. L., Maxwell, D. S. & Tirado-Rives, J. Development and testing of the OPLS all-atom force field on conformational energetics and properties of organic liquids. *J. Am. Chem. Soc.* **118**, 11225–11236, DOI: https://doi.org/10.1021/ja9621760 (1996).

15. Bussi, G., Donadio, D. & Parrinello, M. Canonical sampling through velocity rescaling. *J. Chem. Phys.* **126**, 014101, DOI: https://doi.org/10.1063/1.2408420 (2007).

16. Martyna, G. J. & Tuckerman, M. E. A reciprocal space based method for treating long range interactions in ab initio and force-field-based calculations in clusters. *J. Chem. Phys.* **110**, 2810–2821, DOI: https://doi.org/10.1063/1.477923 (1999).

17. Prendergast, D. & Galli, G. X-ray absorption spectra of water from first principles calculations. *Phys. Rev. Lett.* **96**, 215502, DOI: https://doi.org/10.1103/PhysRevLett.96.215502 (2006).

18. Iannuzzi, M. & Hutter, J. Inner-shell spectroscopy by the Gaussian and augmented plane wave method. *Phys. Chem. Chem. Phys.* **9**, 1599–1610, DOI: 10.1039/B615522G (2007).

19. Krishnan, R., Binkley, J. S., Seeger, R. & Pople, J. A. Self-consistent molecular orbital methods. XX. A basis set for correlated wave functions. *J. Chem. Phys.* **72**, 650–654, DOI: https://doi.org/10.1063/1.438955 (1980).

20. Frisch, M. J., Pople, J. A. & Binkley, J. S. Self-consistent molecular orbital methods 25. Supplementary functions for Gaussian basis sets. *J. Chem. Phys.* **80**, 3265–3269, DOI: https://doi.org/10.1063/1.447079 (1984).

21. Frisch, M. J. *et al.* Gaussian 16 Revision B.01. https://gaussian.com/relnotes_b01/ (2016). Gaussian Inc. Wallingford CT.

22. Møller, C. & Plesset, M. S. Note on an approximation treatment for many-electron systems. *Phys. Rev.* **46**, 618, DOI: https://doi.org/10.1103/PhysRev.46.618 (1934).

23. Frisch, M. J., Head-Gordon, M. & Pople, J. A. A direct MP2 gradient method. *Chem. Phys. Lett.* **166**, 275–280, DOI: https://doi.org/10.1016/0009-2614(90)80029-D (1990).

24. Frisch, M. J., Head-Gordon, M. & Pople, J. A. Semi-direct algorithms for the MP2 energy and gradient. *Chem. Phys. letters* **166**, 281–289, DOI: https://doi.org/10.1016/0009-2614(90)80030-H (1990).

25. Head-Gordon, M., Pople, J. A. & Frisch, M. J. MP2 energy evaluation by direct methods. *Chem. Phys. Lett.* **153**, 503–506, DOI: https://doi.org/10.1016/0009-2614(88)85250-3 (1988).

26. Sæbø, S. & Almlöf, J. Avoiding the integral storage bottleneck in LCAO calculations of electron correlation. *Chem. Phys. Lett.* **154**, 83–89, DOI: https://doi.org/10.1016/0009-2614(89)87442-1 (1989).

27. Head-Gordon, M. & Head-Gordon, T. Analytic MP2 frequencies without fifth-order storage. Theory and application to bifurcated hydrogen bonds in the water hexamer. *Chem. Phys. Lett.* **220**, 122–128, DOI: https://doi.org/10.1016/0009-2614(94)00116-2 (1994).

28. Dunning, T. H. Gaussian basis sets for use in correlated molecular calculations. I. The atoms boron through neon and hydrogen. *J. Chem. Phys.* **90**, 1007–1023, DOI: https://doi.org/10.1063/1.456153 (1989).

29. Kendall, R. A., Dunning, T. H. & Harrison, R. J. Electron affinities of the first-row atoms revisited. Systematic basis sets and wave functions. *J. Chem. Phys.* **96**, 6796–6806, DOI: https://doi.org/10.1063/1.462569 (1992).

30. Peterson, K. A., Woon, D. E. & Dunning, T. H. Benchmark calculations with correlated molecular wave functions. IV. The classical barrier height of the H+ H2→ H2+ H reaction. *J. Chem. physics* **100**, 7410–7415, DOI: https://doi.org/10.1063/1.466884 (1994).

31. Boys, S. F. & Bernardi, F. The calculation of small molecular interactions by the differences of separate total energies. Some procedures with reduced errors. *Mol. Phys.* **19**, 553–566, DOI: https://doi.org/10.1080/00268977000101561 (1970).

32. Simon, S., Duran, M. & Dannenberg, J. How does basis set superposition error change the potential surfaces for hydrogen-bonded dimers? *J. Chem. Phys.* **105**, 11024–11031, DOI: https://doi.org/10.1063/1.472902 (1996).

33. Wormit, M. *et al.* Investigating excited electronic states using the algebraic diagrammatic construction (ADC) approach of the polarisation propagator. *Mol. Phys.* **112**, 774–784, DOI: https://doi.org/10.1080/00268976.2013.859313 (2014).

34. Shao, Y. *et al.* Advances in molecular quantum chemistry contained in the Q-Chem 4 program package. *Mol. Phys.* **113**, 184–215, DOI: https://doi.org/10.1080/00268976.2014.952696 (2015).

35. Wenzel, J., Wormit, M. & Dreuw, A. Calculating core-level excitations and x-ray absorption spectra of medium-sized closed-shell molecules with the algebraic-diagrammatic construction scheme for the polarization propagator. *J. Comput. Chem.* **35**, 1900–1915, DOI: https://doi.org/10.1002/jcc.23703 (2014).

36. Wenzel, J., Holzer, A., Wormit, M. & Dreuw, A. Analysis and comparison of CVS-ADC approaches up to third order for the calculation of core-excited states. *J. Chem. Phys.* **142**, 214104, DOI: https://doi.org/10.1063/1.4921841 (2015).

37. Wenzel, J. & Dreuw, A. Physical properties, exciton analysis, and visualization of core-excited states: An intermediate state representation approach. *J. Chem. Theory Comput.* **12**, 1314–1330, DOI: https://doi.org/10.1021/acs.jctc.5b01161 (2016).

38. Clark, T., Chandrasekhar, J., Spitznagel, G. W. & Schleyer, P. V. R. Efficient diffuse function-augmented basis sets for anion calculations. III. The 3-21+ G basis set for first-row elements, Li–F. *J. Comput. Chem.* **4**, 294–301, DOI: https://doi.org/10.1002/jcc.540040303 (1983).

39. Vaz da Cruz, V. *et al.* Nuclear dynamics in resonant inelastic X-ray scattering and X-ray absorption of methanol. *J. Chem. Phys.* **150**, 234301, DOI: https://doi.org/10.1063/1.5092174 (2019).

40. Runge, E. & Gross, E. K. U. Density-functional theory for time-dependent systems. *Phys. Rev. Lett.* **52**, 997, DOI: https://doi.org/10.1103/PhysRevLett.52.997 (1984).

41. Becke, A. D. Density-functional thermochemistry. III. The role of exact exchange. *J. Chem. Phys.* **98**, 5648–5652, DOI: https://doi.org/10.1063/1.464913 (1993).

42. Dunlap, B. I., Connolly, J. & Sabin, J. On some approximations in applications of Xα theory. *J. Chem. Phys.* **71**, 3396–3402, DOI: https://doi.org/10.1063/1.438728 (1979).

43. Van Alsenoy, C. Ab initio calculations on large molecules: The multiplicative integral approximation. *J. Comput. Chem.* **9**, 620–626, DOI: https://doi.org/10.1002/jcc.540090607 (1988).

44. Kendall, R. A. & Früchtl, H. A. The impact of the resolution of the identity approximate integral method on modern ab initio algorithm development. *Theor. Chem. Accounts* **97**, 158–163, DOI: https://doi.org/10.1007/s002140050249 (1997).

45. Eichkorn, K., Treutler, O., Öhm, H., Häser, M. & Ahlrichs, R. Auxiliary basis sets to approximate Coulomb potentials. *Chem. Phys. Lett.* **240**, 283–290, DOI: https://doi.org/10.1016/0009-2614(95)00621-A (1995).

46. Eichkorn, K., Weigend, F., Treutler, O. & Ahlrichs, R. Auxiliary basis sets for main row atoms and transition metals and their use to approximate Coulomb potentials. *Theor. Chem. Accounts* **97**, 119–124, DOI: https://doi.org/10.1007/s002140050244 (1997).

47. Whitten, J. L. Coulombic potential energy integrals and approximations. *J. Chem. Phys.* **58**, 4496–4501, DOI: https://doi.org/10.1063/1.1679012 (1973).

48. Weigend, F., Köhn, A. & Hättig, C. Efficient use of the correlation consistent basis sets in resolution of the identity MP2 calculations. *J. Chem. Phys.* **116**, 3175–3183, DOI: https://doi.org/10.1063/1.1445115 (2002).

49. Neese, F. The ORCA program system. *Wiley Interdiscip. Rev. Comput. Mol. Sci.* **2**, 73–78, DOI: https://doi.org/10.1002/wcms.81 (2012).

50. Neese, F. Software update: the ORCA program system, version 4.0. *Wiley Interdiscip. Rev. Comput. Mol. Sci.* **8**, e1327, DOI: https://doi.org/10.1002/wcms.1327 (2018).

51. Tokushima, T. *et al.* Selective observation of the two oxygen atoms at different sites in the carboxyl group (-COOH) of liquid acetic acid. *Phys. Chem. Chem. Phys.* **11**, 1679–1682, DOI: https://doi.org/10.1039/B818812B (2009).

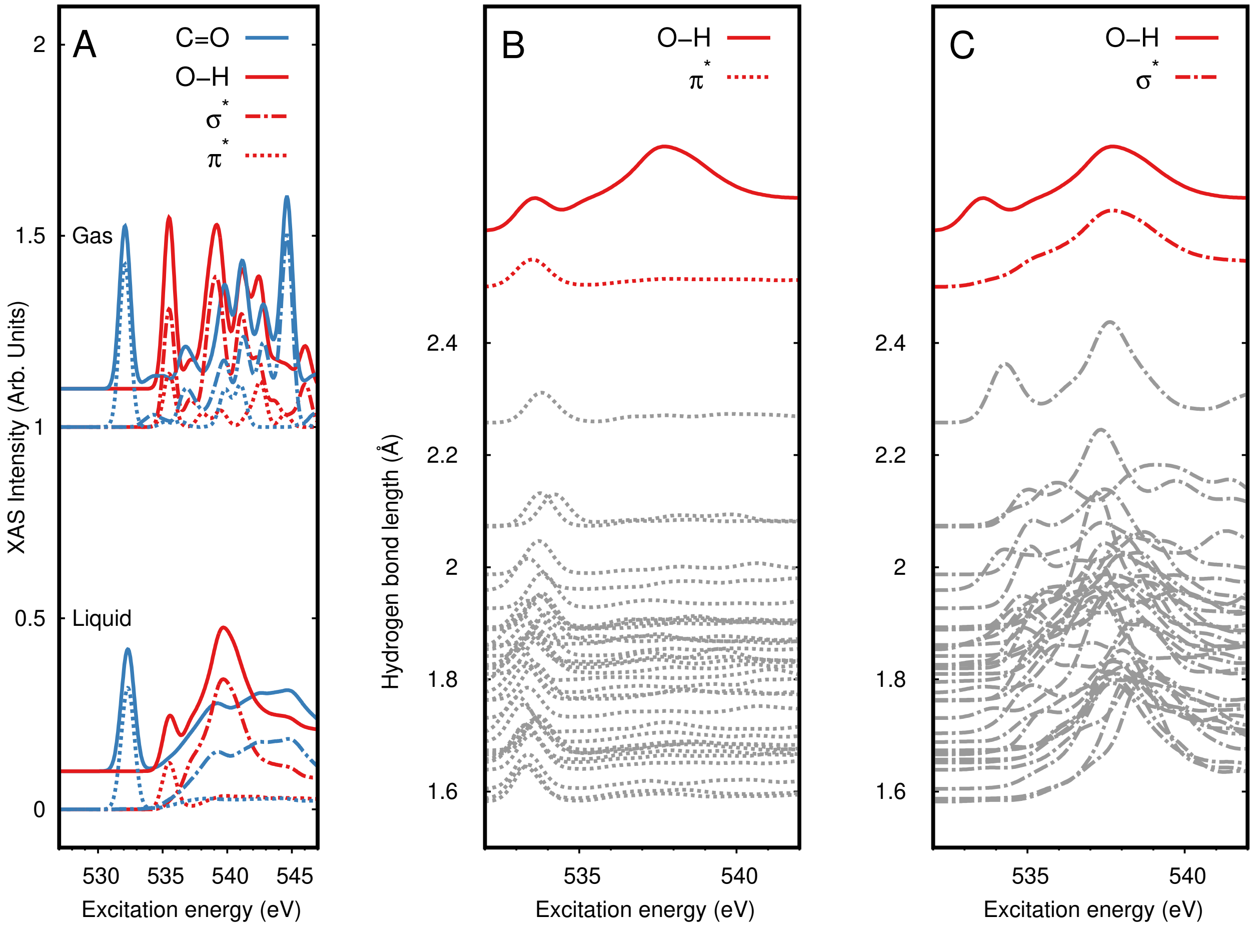

Figure S1. Simulated oxygen K-edge x-ray absorption spectra (XAS) of liquid acetic acid from DFT using core-excited state (XFH) transition potential calculations[17]. (a) The XAS of the O_C (blue) and O_H (red) oxygen atoms of gas and liquid ACA are shown and further decomposed into out-of-plane (π^* - dotted) and in-plane (σ^* - dashed-dotted) contributions. (b) Hydrogen bond distance (H $\cdots$ O) dependence of the π^* excitation contribution in 1s O_H XAS of individual sampled configurations (gray). The individual contributions are computed for the angle between the donating OH group and nearest oxygen atom larger than 150° (c) The same as in (b) for the σ^* excitation contribution.

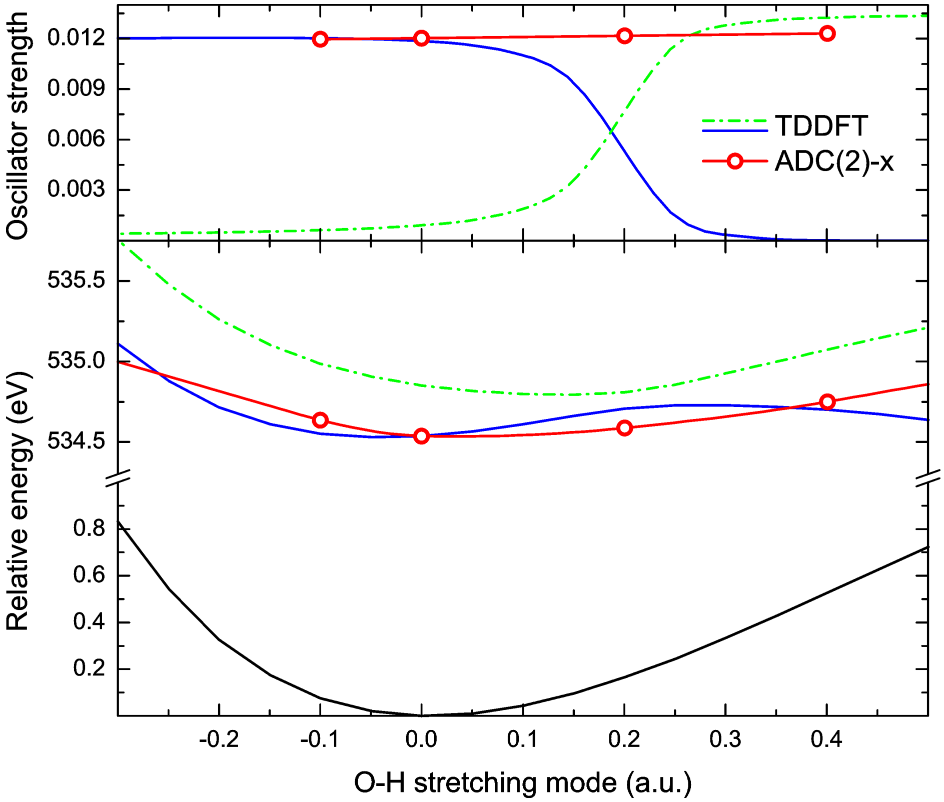

Figure S2. The potential energy curves (lower panel) and oscillator strengths (upper panel) for the inverted dimer computed using TDDFT (blue and green lines) and CVS-ADC(2)-x (red line) for scattering through the $O_H 1s^{-1}\pi^*$ core-excited states of the "in" oxygen. The PEC based on the TDDFT method has been shifted to match the PEC obtained using the CVS-ADC(2)-x technique at the point of the ground state equilibrium (it is shifted up by 15 eV). The black solid line shows the ground state PEC.

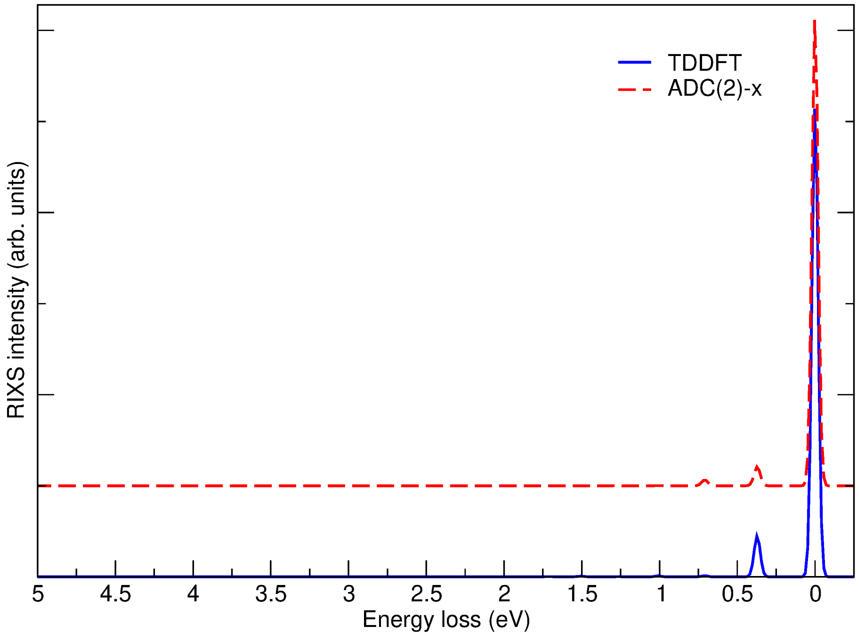

Figure S3. The RIXS profile for the inverted dimer computed using the PECs obtained with TDDFT and CVS-ADC(2)-x (see Fig. S2). The scattering through the $O_H 1s^{-1}\pi^*$ core-excited states is calculated taking into account the R-dependence of the transition dipole moment.

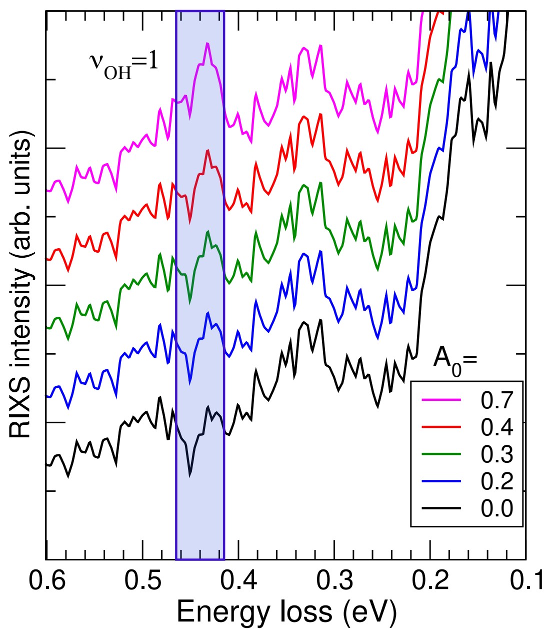

Figure S4. Averaged RIXS spectrum measured at five excitation energies near the top of the O_H pre-edge resonance (534.1, 534.4, 534.7, 535.0, 535.3 eV) superimposed with a Gaussian function of FWHM=0.05 eV centered at 0.44 eV energy loss in order to mimic the $\nu_{OH} = 1$ vibrational resonance. The peak intensity A_0 of the Gaussian function is varied from 0.0 to 0.7 (see legends); the curves for different A_0 are shifted vertically for clarity.

.

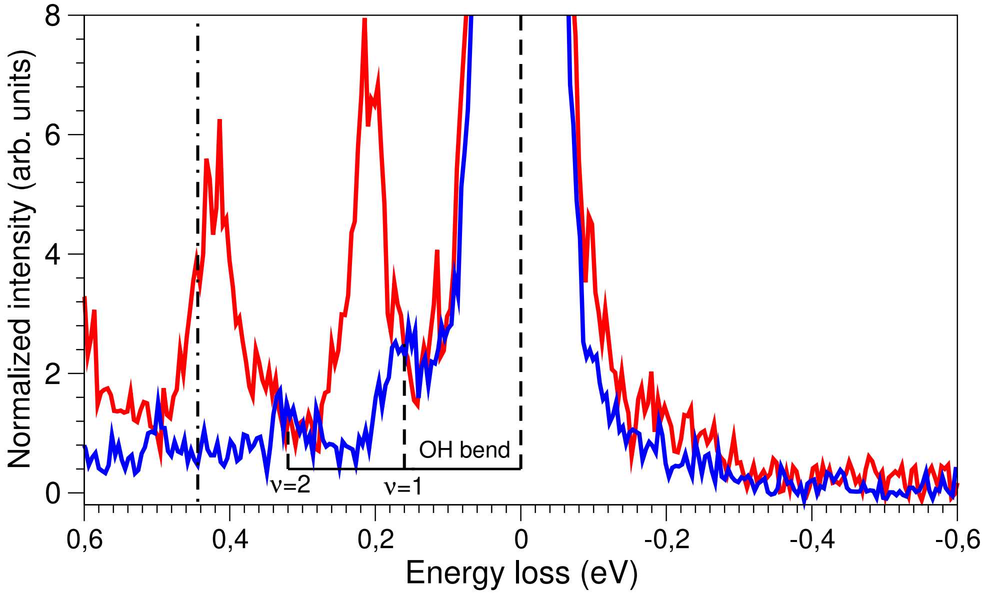

Figure S5. Detailed view of RIXS measured at the O_C pre-edge resonance (red) and at the O_H pre-edge resonance (blue) of liquid ACA. The intensities of O_C and O_H RIXS spectra are matched to have the same noise (the root mean square error) level in the anti-Stockes part of the spectra -2.0. . . -6.0 eV. See also the main text.

.

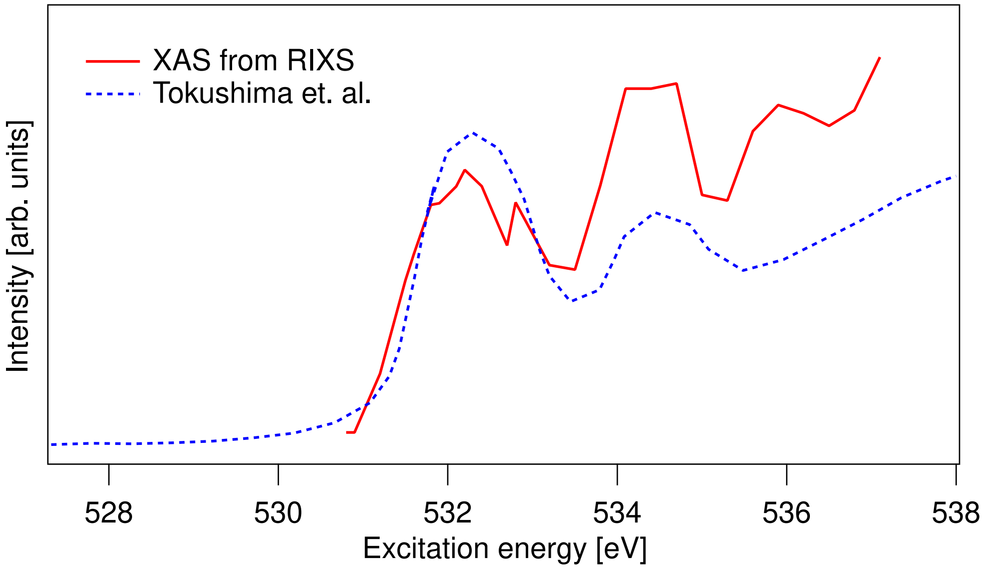

Figure S6. Approximate XAS obtained from our measurements as the RIXS intensity integrated in a broad energy range (up to 12 eV energy loss) at several values of the photon energies across the O pre-edge of ACA. The XAS measurements from the literature[51] is given for comparison.
